# Peer Review of “Angiotensin Converting Enzyme 1 Expression in the Leukocytes of Adults Aged 64 to 67 Years”

**DOI:** 10.2196/45279

**Published:** 2023-01-20

**Authors:** Calogero Caruso

**Affiliations:** 1 University of Palermo Palermo Italy

**Keywords:** aging, angiotensin converting enzyme, lymphocytes, immunosenescence, inflammaging

*This is a peer-review report submitted for the paper “Angiotensin Converting Enzyme 1 Expression in the Leukocytes of Adults Aged 64 to 67 Years.”* [1]

## Round 1 Review

### General Comments

The paper is essentially anecdotal because it studies the cells of 6 subjects without any comparison with other age groups. There is also a serious limitation because beyond the age and sex, there is no information on the donors (how and why they were recruited, what drugs they took, etc). To infer that chronological and biological ages do not match is inappropriate in the absence of the above information.

However, the paper is of some interest because there are few studies on the topic.

### Specific Comments

Essential revisions that are required to verify the manuscript

Although we do not have data on donors, placing an age and gender column in all tables adds a minimum of useful information for the reader.

Inflammaging means low grade of inflammation. The CRP value of 23.1 suggests acute inflammation (also because albumin has high values, while in chronic inflammation its values decrease). Therefore the averages do not have to take this subject into account.

Other suggestions to improve the manuscript

The authors write that their findings suggest that ACE1 could play a role in several processes linked to aging including the generation and activation of autoimmune cells, due to the experimental evidence that inhibitors of ACE suppress the autoimmune process in a number of autoimmune diseases such as EAE, arthritis, autoimmune myocarditis [2]. They do not appear to have these findings in their paper. So, they need to change the sentence.

### Decision

Requires revisions: The manuscript contains objective errors or fundamental flaws that must be addressed and major revisions are suggested.

### Decision Changed

Verified manuscript: The content is scientifically sound, only minor amendments (if any) are suggested.

## Round 2 Review

### Decision Changed

Verified manuscript: The content is scientifically sound, and only minor amendments (if any) are suggested.
